# PBMC-Humanized Mouse Model for Multiple Sclerosis: Studying Immune Changes and CNS Involvement

**DOI:** 10.21769/BioProtoc.5312

**Published:** 2025-05-20

**Authors:** Anastasia Dagkonaki, Irini Papazian, Vasileios Gouzouasis, Ariadni Karles, Maria Kourouvani, Theodore Tselios, Eirini Fragkiadaki, Lesley Probert

**Affiliations:** 1 Laboratory of Molecular Genetics, Department of Immunology, Hellenic Pasteur Institute, Athens, Greece; 2 Department of Animal Models for Biomedical Research, Hellenic Pasteur Institute, Athens, Greece; 3 Department of Chemistry, University of Patras, Patras, Greece

**Keywords:** Humanized mice, B2m-NOG mice, PBMC engraftment, Multiple sclerosis, Experimental autoimmune encephalomyelitis, Epstein–Barr virus infection status

## Abstract

Humanized immune system (HIS) mice are powerful tools for studying human immune system function and dysfunction and developing human-specific immunotherapeutics. The availability of sophisticated super immunodeficient mouse strains has allowed immune system humanization using transplants of human peripheral blood mononuclear cells (PBMC) or hematopoietic stem cells. HIS mice are used extensively in immune-oncology, while there are fewer studies in autoimmunity, especially multiple sclerosis (MS). Using the protocol described here, we generated HIS mice that show key features of MS not represented in other widely used MS models [1]. Severely immunodeficient NOD.Cg-*B2m^em1Tac^ Prkdc^scid^ Il2rg^tm1Sug^*/JicTac (B2m-NOG) mice, which lack murine B, T, and NK cells and murine major histocompatibility class I molecules and have defective innate immune responses, were transplanted with PBMC from HLA-DRB1-genotyped MS patients and healthy donors. Mice were successfully engrafted with hCD4 and hCD8 T and B lymphocytes and developed both spontaneous and experimental autoimmune encephalomyelitis (EAE)-enhanced T-cell lesions in the central nervous system. B-cell engraftment was highest in mice receiving cells from MS patients with serological evidence for Epstein–Barr virus (EBV) reactivation. This humanized MS model shows advantages over EAE, particularly spontaneous hCD8 T-cell lesions in the brain and spinal cord, mixed hCD8/hCD4 T-cell lesions in EAE-immunized mice, and more severe lesions in mice engrafted with PBMC from MS donors carrying the DRB1*15 MS susceptibility allele compared to DRB1*15-positive healthy and DRB1*13-positive MS donors. MS HIS mice represent simple and rapid tools for investigating human immunopathology and the efficacy of therapeutics at a personalized level.

Key features

• Humanization of severely immunodeficient B2m-NOG mice with human PBMC.

• Engraftment analysis of human immune system in mice using multicolor flow cytometry.

• Animal familiarization and handling techniques.

• Epstein-Barr virus latency evaluation in human plasma.

• Ex vivo characterization of engrafted T-cell cytokine responses.

## Graphical overview


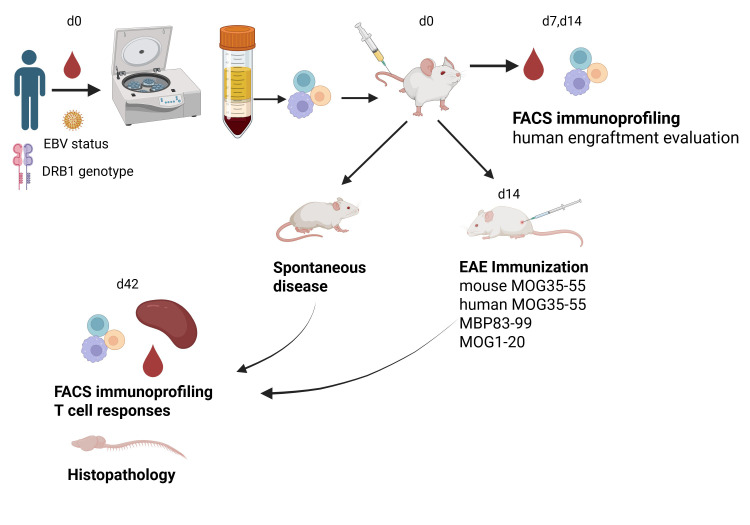


## Background

Multiple sclerosis (MS) is a chronic demyelinating disease of the central nervous system (CNS) that shows autoimmune characteristics and causes progressive neurodegeneration [2]. Disease modeling for experimental studies is a major challenge. A widely used model is experimental autoimmune encephalomyelitis (EAE), induced in rodents by immunization with myelin antigens and resulting in T cell–mediated CNS inflammation and demyelination [3,4]. EAE and MS share many clinical, immunological, and pathological similarities [5,6] but also present significant differences [7,8], as follows: 1) the T cell–dependent autoantigens in EAE are known, whereas in MS they are not well defined [9,10]; 2) there is a predominance of CD8+ T cells in MS lesions, while these cells are rare in EAE [11]; 3) inflammation in EAE primarily targets the spinal cord, whereas in MS it mainly targets the brain; 4) most EAE models develop independently of B cells, while B-cell depleting therapies are highly successful in MS [12]; 5) EAE mice cannot be infected with EBV, which is a prerequisite for MS development [13]; and 6) many drugs showing high efficacy in EAE are not useful for MS [14]. Previous attempts to develop a humanized MS model using HIS mice included mice transplanted with peripheral blood mononuclear cells (PBMCs) from healthy individuals and immunized for EAE [15], mice transplanted with hematopoietic stem cells derived from DRB1*15-positive and negative donors and infected with EBV [16], and mice transplanted with PBMCs from individuals with prior Epstein–Barr virus (EBV) infection and/or relapsing remitting MS (RRMS) [17]. While the available MS models recapitulate certain aspects of the human disease [18], there remains a pressing need for improved models of MS for studying the full spectrum of human immune responses and evaluating the therapeutic efficacy of novel cellular and molecular targets.

Here, we describe a simple and rapid procedure for the generation of a novel humanized model for MS [1]. We provide detailed protocols for PBMC isolation from human peripheral blood, characterization of EBV latency status in donor plasma, monitoring of human immune cell engraftment by flow cytometry, and an immunization protocol for EAE induction. Briefly, freshly isolated PBMCs from MS and healthy control individuals were transplanted into severely immunodeficient B2m-NOG mice, resulting in progressive engraftment by human CD4 and CD8 T cells and B cells, with significant variability between different PBMC donors. In agreement with previous studies, the lack of mouse MHC class I molecules in B2m-NOG mice, due to β2-microglobulin gene deletion (*B2m*), allowed preferential engraftment of CD4 T cells over CD8 T cells and delayed the onset of xenogeneic graft versus host disease (GVHD) [1,19]. PBMC transplants from donors with recent/ongoing EBV reactivation showed high human B-cell engraftment. Importantly, PBMC transplants from MS patients carrying an HLA-DRB1*15:01 MS susceptibility allele developed spontaneous and EAE-induced CD8 and CD4 T-cell lesions in the brain and spinal cord, a finding that supports a pathogenic role of T cells in MS [1]. We describe the major limitations of the current model that require further development to achieve more complete modeling of MS in humanized mice.

## Materials and reagents

**Biological materials**

1. Fresh ethylenediaminetetraacetic acid (EDTA)-treated peripheral blood from human individuals pre-screened for pathogenic viruses and HLA-DRB1-genotyped

2. B2m-NOD/Shi—scid IL2rg^null^ (B2m-NOG) mice (stock number 14,957 F: NOD.Cg-B2m^em1Tac^Prkdc^scid^Il2rg ^tm1Sug^/JicTac; Taconic Biosciences)

**Reagents**

1. Ficoll-Histopaque^®^-1077 (Sigma-Aldrich, catalog number: 10771)

2. Fetal bovine serum (FBS) (Gibco, catalog number: 10270106)

3. L-Glutamine (Gibco, catalog number: 25030024)

4. Penicillin/streptomycin (Gibco, catalog number: P4333)

5. RPMI-1640 (Sigma-Aldrich, catalog number: R1145)

6. Quicklysis erythrocyte lysis buffer (Cytognos, catalog number: CYT-QL-1)

7. Hanks’ balanced salt solution (HBSS) (Gibco, catalog number: 14175053)

8. Freund’s complete adjuvant (FCA) (Sigma-Aldrich, catalog number: F5881)

9. H37Ra *Mycobacterium tuberculosis* (desiccated) (BD^TM^ Difco^TM^, catalog number: 231141)

10. *Bordetella pertussis* toxin (PTx) (Sigma-Aldrich, catalog number: P2980)

11. Phorbol 12-myristate 13-acetate (PMA) (Sigma-Aldrich, catalog number: P1585)

12. Ionomycin (Sigma-Aldrich, catalog number: I0634)

13. Brefeldin-A (Sigma-Aldrich, catalog number: B7651)

14. 2-β-mercaptoethanol (AnalaR, catalog number: 10459)

15. BV-510 anti-human CD45 (BioLegend, clone 2D1, catalog number: 368526)

16. APC-Cyanine 7 anti-human CD3 (BioLegend, clone HIT3a, catalog number: 300318)

17. PE-Cyanine 7 anti-human CD4 (BioLegend, clone A161A1, catalog number: 357410)

18. PerCP anti-human CD8 (BioLegend, clone SK1, catalog number: 344708)

19. PE anti-human CD19 (BioLegend, clone 4G7, catalog number: 392506)

20. FITC anti-human CD56 (BioLegend, clone 5.1H11, catalog number: 362546)

21. APC anti-mouse CD45 (BioLegend, clone 30-F11, catalog number: 103112)

22. BV-421 anti-human CD14 (BioLegend, clone HCD14, catalog number: 325628)

23. APC anti-human CD66b (BioLegend, clone G1OF5, catalog number: 305117)

24. APC anti-human CD4 (BD Biosciences, clone RPA-T4, catalog number: 555349)

25. PE-IFN-γ anti-human (BD Biosciences, clone B27, catalog number: 559327)

26. PE-IL-17A anti-human (eBioscience, clone eBio64DEC17, catalog number: 12–7179-42)

27. EDTA, disodium salt 2-hydrate (Applichem, catalog number: 131669.1210)

28. NaCl (Applichem, catalog number: 131659.1211)

29. KCl (BDH, AnalaR, catalog number: 101984L)

30. Na_2_HPO_4_ (Applichem, catalog number: 131679.1210)

31. KH_2_PO_4_ (Applichem, catalog number: A2946,1000)

32. Saponin (Applichem, catalog number: A2542,1000)

33. NH_4_Cl (BDH, AnalaR, catalog number: 100173D)

34. Glucose (Sigma-Aldrich, catalog number: G7021)

35. Phenol red (BDH, AnalaR, catalog number: 200913V)

36. MgCl_2_6H_2_O (BDH, AnalaR, catalog number: 101494V)

37. MgSO_4_7H_2_O (Scharlau, catalog number: MA00860500)

38. CaCl_2_2H_2_O (BDH, AnalaR, catalog number: 100704Y)

39. NaHCO_3_ (BDH, AnalaR, catalog number: 102474V)

40. NaOH (BDH, AnalaR, catalog number: 28244.295)

41. KHCO_3_ (Honeywell, catalog number: 12602)

42. NaHCO_3_ 7.5% (Biosera, catalog number: LM-S2046/100)

43. Bovine serum albumin (BSA) (Sigma-Aldrich, catalog number: A9418)

44. Paraformaldehyde (PFA) (Sigma-Aldrich, catalog number: 158127–5G)

45. 0.9% NaCl solution (saline) (Demo, catalog number: 5205411002291)

46. Heparin sodium 5000 i.u./mL solution (Leo Pharma, registration number: BR-1005)

47. Myelin peptides MBP83–99, MOG1–20, mMOG35–55, and hMOG35–55 (synthesized as previously described [20]).

48. ELISA-VIDITEST anti-EBNA-1 EBV IgG (VIDIA, catalog number: ODZ-001)

49. ELISA-VIDITEST anti-EBNA-1 EBV IgM (VIDIA, catalog number: ODZ-002)

50. ELISA-VIDITEST anti-EA(D) EBV IgG (VIDIA, catalog number: ODZ-006)

51. ELISA-VIDITEST anti-VCA EBV IgG (VIDIA, catalog number: ODZ-265)

52. ELISA-VIDITEST anti-VCA EBV IgM (VIDIA, catalog number: ODZ-005)

53. ELISA-VIDITEST anti-VCA EBV IgA (VIDIA, catalog number: ODZ-096)

**Solutions**

1. EDTA 0.5 M (pH 8) (see Recipes)

2. Phosphate-buffered saline (PBS) (see Recipes)

3. Permeabilization buffer (see Recipes)

4. Gey’s erythrocyte lysis (see Recipes)

5. 4% paraformaldehyde (PFA) (see Recipes)

6. RPMI supplemented (see Recipes)

7. ACK solution (see Recipes)

**Recipes**

**1. EDTA 0.5 M (pH 8)**

*Note: Initial water volume should be 200–300 mL as EDTA will take a substantial volume when fully dissolved. Allow the solution to mix completely before adding any more water.

**Table:** 

Reagent	Final concentration	Quantity or Volume
EDTA	0.5 M	93.1 g
NaOH	20 g/L	10 g
H_2_O	n/a	see note*
Total	n/a	500 mL

**2. Phosphate-buffered saline (PBS)**

*Note: Initial water volume should be 400 mL as salts will take a substantial volume when fully dissolved. Allow the solution to mix completely before adding any more water. pH should be adjusted to 7.4 using drops of 4 M NaOH.

**Table:** 

Reagent	Final concentration	Quantity or Volume
NaCl	8 g/L	4 g
KCl	0.2 g/L	0.1 g
Na_2_HPO_4_	0.6 g/L	0.3 g
KH_2_PO_4_	0.2 g/L	0.1 g
H_2_O	n/a	see note*
Total	n/a	500 mL

**3. Permeabilization buffer**

**Table:** 

Reagent	Final concentration	Quantity or Volume
Saponin	0.5% w/v	0.25 g
BSA	0.5% w/v	0.25 g
PBS	n/a	50 mL
Total	n/a	50 mL

**4. Gey’s erythrocyte lysis**

This reagent consists of three different solutions (A, B, and C).

**Table:** 

Solution A	Final concentration	Quantity or Volume
NH_4_Cl	35 g/L	17.5 g
KCl	1.85 g/L	0.925 g
Na_2_HPO_4_	1.58 g/L	0.79 g
KH_2_PO_4_	0.12 g/L	0.06 g
Glucose	5 g/L	2.5 g
Phenol red	5 mg/L	25 mg
H_2_O	n/a	To 500 mL
Total	n/a	500 mL

**Table:** 

Solution B	Final concentration	Quantity or Volume
MgCl_2_6H_2_O	4.2 g/L	0.42 g
MgSO_4_7H_2_O	1.4 g/L	0.14 g
CaCl_2_2H_2_O	3.1 g/L	0.31 g
H_2_O	n/a	To 100 mL
Total	n/a	100 mL

**Table:** 

Solution C	Final concentration	Quantity or Volume
NaHCO_3_	22.5 g/L	2.25 g
H_2_O	n/a	To 100 mL
Total	n/a	100 mL

**Table:** 

Gey’s Solution	Final concentration	Quantity or Volume
Solution A	n/a	40 mL
Solution B	n/a	10 mL
Solution C	n/a	10 mL
H_2_O	n/a	140 mL
Total	n/a	200 mL

**5. 4% paraformaldehyde (PFA)**

*Note: Initial PBS volume should be 380 mL as PFA will take a substantial volume when fully dissolved. Allow the solution to mix completely before adding any more PBS. pH should be adjusted to 7.4.

**Table:** 

Reagent	Final concentration	Quantity or Volume
PFA	4% w/v	20 g
PBS	n/a	380 mL
NaOH 4M	n/a	200 μL (*see note)
Total	n/a	500 mL

**6. RPMI supplemented**

*Note: Addition of NaOH solution is for pH adjustment. The solution must be added 500 μL each time, as the final volume may vary.

**Table:** 

Reagent	Final concentration	Quantity or Volume
RPMI-1640 (10×)	n/a	50 mL
FBS	10% v/v	50 mL
L-Glutamine	1% v/v	5 mL
Penicillin/streptomycin	1% v/v	5 mL
Sodium bicarbonate 7.5%	n/a	14 ml
NaOH solution 4 M	n/a	1.5 mL (see note*)
2-β-mercaptoethanol	n/a	3 μL
H_2_O	n/a	374.5 mL
Total	n/a	500 mL

**7. ACK solution**

*Note: pH should be adjusted to 7.4.

**Table:** 

Reagent	Final concentration	Quantity or Volume
NH_4_Cl	0.15 M	4.3 g
KHCO_3_	10 mM	0.5 g
EDTA	0.1 mM	100 μL of 0.5 EDTA solution
H_2_O	n/a	500 mL
Total	n/a	500 mL

**Laboratory supplies**

1. 50 mL centrifuge polypropylene tubes (Greiner, catalog number: 227261)

2. 15 mL centrifuge polypropylene tubes (Greiner, catalog number: 188261)

3. 25 mL polystyrene pipettes (Sarstedt, catalog number: 86.1685.001)

4. 10 mL polystyrene pipettes (Sarstedt, catalog number: 86.1254.001)

5. Petri dishes 94 ×16 mm (Greiner, catalog number: 632180)

6. 6-well plate (Corning^TM^, catalog number: 3516)

7. 70 μm cell strainer (Corning^TM^, catalog number: 431751)

8. 1.5 mL microcentrifuge polypropylene tubes (Greiner, catalog number: 616201)

9. 7 mL (bijou) polystyrene container (Greiner Bio-One, catalog number: 189171)

10. 200 μL pipette tips (Greiner Bio-One, catalog number: 740290)

11. 1000 μL pipette Tips (Greiner Bio-One, catalog number: 739290)

12. Micro-fine insulin syringe 1 mL 29G × 12.7 mm (BD, catalog number: 324825)

13. Falcon^®^ round-bottom polystyrene tubes, 5 mL (flow cytometry tubes) (Corning, catalog number: 352052)

14. Neubauer chamber for cell counting (HBG Germany, catalog number: HP2010103)

15. Mouse restrainer (Plas Labs, catalog number: 551-BSRR)

16. Retort Stand Set Complete kit with stand and 3-prong clamp (Camlab, catalog number: 1177157)

17. Syringe needle 27G × 1/2" (BD Microlance, catalog number: 300635)

18. Glass Dounce homogenizer and pestle (DWK Life Sciences, catalog number: 358103)

19. Glass pipettes 230 mm (Deltalab, catalog number: 712)

20. Cryogenic vial (Corning, catalog number: 430659)

21. Vacutainer EDTA 10 mL (optional) (BD, catalog number: 367525)

**Animal maintenance supplies**

1. Standard pelleted feed for rodents (Mucendola Sri, Italy, catalog number: 4RF21-GLP)

2. Irradiated treats and enrichment materials for rodents (Carfil Quality, Belgium)

3. Autoclavable corn-cob bedding for IVC cages (Scobis-Due, Mucendola Sri, Italy)

4. Water-gels for rodents (Solid-Drink, Triple-A-Trading, Netherlands)

## Equipment

1. Megafuge 16R refrigerated centrifuge (Thermo Fisher Scientific, model: 75004271)

2. Microcentrifuge (Eppendorf, model: 5430 R)

3. BSL-2 biosafety cabinet for human cells (Bioair, catalog number/model: LDC0000/TopSafe 1.8)

4. BSL-2 laminar flow cabinet for mouse cells (Holten Laminair, model: HB 2472)

5. Pipette gun (Gilson, model: Gilson Macroman PGI110120)

6. Pipette set (Gilson, model: Gilson Pipetman F144056M, F144058M, F144059M)

7. Waterbath (Grant Instruments, model: JB1)

8. FACSMelody cytometer (BD, catalog number: 653885) or a similar analyzer with the minimum requirement of three lasers: a violet, blue, and red laser for the suggested antibody panels mentioned in sections E, F, and J

9. FACSCalibur cytometer (BD, catalog number: 342975) or a similar analyzer with the minimum requirement of two lasers: blue and red for the suggested antibody panels mentioned in section K

10. Spectrophotometer/colorimeter (microplate reader) (Tecan, model: Spark^®^)

11. Fiber optic light source (Nikon, model: 72306)

12. Infrared thermal lamp 275 W (Philips, catalog number: 101637)

13. Ventilation unit for individually ventilated cages-IVC (Ehret Bio A.S., catalog number: 362-THF3365)

14. Racks for individually ventilated cages (IVC) (Ehret Bio A.S., catalog number: 362-THF-XXX)

15. Individually ventilated cages-IVC for mice (Bio A.S Cages Type IL, catalog number: 362-CILV-XXX)

16. BSL-2 laminar flow cabinet for animals (ESI Flufrance, model: 12461)

17. Ultrasonic homogenizer (Cole Parmer, model: 4710 series)

18. Shaving machine (Philips, model: Hairclipper series 3000, model: HC3525/15)

## Software and datasets

1. FlowJo software (Tree Star, Inc, V10)

2. Microsoft Excel (Microsoft, 2016)

3. GraphPad Prism 6.01 (GraphPad, 2012)

## Procedure

**A. Animal handling**

The described animal handling procedures are specifically necessary for mice of the B2m-NOG strain compared to other common mouse strains to avoid their tendency to develop high stress and excitability during the experimental procedures.

1. Mice are maintained in individually ventilated cage (IVC) systems to safeguard and maintain their specific pathogen-free (SPF) microbiological status from the commercial supplier. Standard pelleted feed and chlorinated water are provided ad libitum to animals. Animals are housed under a 12/12 h light/dark photoperiod, 22–25 °C temperature, and 35%–75% humidity.

2. Allow mice to acclimatize to the local facility’s conditions for a minimum of seven days. Ensure sterile certified enrichment material (sterile cotton swabs, mouse houses or igloo nests, paper tubes, etc.) in each cage and monitor the normal vital signs and behavior of the mice.

3. Start the human-mouse habituation process, based on a previous report [21,22], by gently cupping each mouse and allowing it to explore freely the experimenter’s palm for 1–3 min daily for 3–5 consecutive days. Since the B2m-NOG strain is extremely immunodeficient, all procedures should be performed inside the BSL-2 laminar flow cabinet for animals. Contact time and duration depend on the behavior of each animal, whose respiratory rate, urination, biting, or escaping efforts are noted and assessed as welfare indicators. The same animal should be handled by the same animal user.

4. Proceed to mouse training for tail blood sampling by gently introducing them to the mouse restrainer, petting and positively reinforcing them by offering them a treat (irradiated sunflower seeds, fruit gels, etc.). This step lasts 3–5 min/mouse/day for 3 days. Afterward, add the tail puncturing step and always monitor the welfare indicators. A total of 6–8 days is required for tail bleeding training.

5. Trained animals are used for the animal study. At the end of each handling and procedure (injections, bleeding, symptom scoring, etc.), mice are offered a treat. For the EAE model, refinement is applied (i.e., soft bedding, water-gels with glucose), and clinical endpoints are monitored daily.

**B. PBMC isolation from human peripheral blood**

For the study using the humanization model described here, PBMCs were isolated from the peripheral blood of six long-term RRMS patients, of which five were HLA-DRB1*15-positive (DR15 MS1–5) and one was HLA-DRB1*13-positive (DR13 MS), all presenting with highly active disease following immunomodulatory treatment with natalizumab. A DR15-positive healthy individual (DR15 HI) was selected as a control donor [1].

**CAUTION:** Human blood donors should be pre-screened for pathogenic viruses, and fresh blood should be handled in a BSL-2 biosafety cabinet.

1. One hour before the blood draw, fill a 50 mL tube with 0.5 M EDTA solution (pH 8) to perform coating. All procedures of this stage must be performed inside the high-level BSL-2 biosafety cabinet.

2. Remove the EDTA solution and leave about 100 μL for each 10 mL of blood. For 50 mL of blood, the tube must contain 500 μL of EDTA solution.

*Note: Blood could alternatively be collected in 10 mL EDTA Vacutainers.*

3. Transfer the fresh EDTA-treated blood at RT on the same day to the laboratory.

4. Keep 5 mL of the whole blood aside for flow cytometry immunoprofiling and plasma isolation.

5. Aliquot the remaining 45 mL of blood into three different 50 mL tubes (15 mL each) and dilute each with an equal volume of PBS.

**Figure 1.**
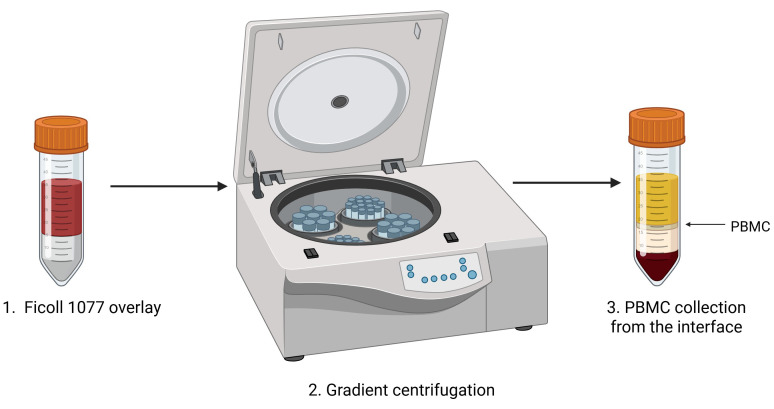
Peripheral blood mononuclear cell (PBMC) isolation protocol. Fresh whole peripheral blood is diluted with an equal volume of PBS, gently layered over Ficoll-Histopaque^®^-1077. The tube is placed in the centrifuge for gradient centrifugation. The PBMC buffy coat is located at the interface of Ficoll and plasma, as indicated (arrow).

*Note: Optimization of the PBMC preparation protocol: Fresh human PBMCs were used for the generation of humanized immune mice using this transplantation protocol [1]. Alternatives include blood diluted 1:1 with RPMI and stored at 4 °C for 72 h prior to PBMC preparation, or fresh PBMCs snap-frozen, stored at -80 °C, thawed, and cultured for 24 h, although these alternative procedures were found to be sub-optimal and showed skewing of immune cell lineage subsets. See Figure 1, Figure supplement 2 of the original research paper [1].*

6. Add 15 mL of Ficoll-Histopaque^®^-1077 to three separate clean 50 mL tubes.

7. Carefully overlay the diluted fresh blood over the Ficoll-Histopaque^®^-1077.

**CAUTION:** In this step, the overlay has to be done very carefully so that the Ficoll does not mix with the blood.

8. Centrifuge at 800× *g* for 25 min at 25 °C (RT), with acceleration: 1 and deceleration: 1.

9. Remove some of the upper “plasma” layer to gain easier access to the buffy coat.

10. Collect the buffy coat and transfer it into separate clean 15 mL tubes (from each 50 mL tube, the buffy coat should be divided into two separate 15 mL tubes). The buffy coat is located at the interface of plasma and Ficoll (Figure 1).

**CAUTION:** Ensure the different phases do not mix.

11. Add 10 mL of 2% FBS/PBS and centrifuge at 300× *g* for 10 min at RT, with acceleration: 7 and deceleration: 7.

12. Remove the supernatant, resuspend the cell pellet in 2 mL of Quicklysis erythrocyte cell lysis buffer, and incubate at RT for 10 min.

*Note: Erythrocyte lysis can alternatively be achieved using ACK solution at RT for 2 min.*

13. Centrifuge: 300× *g*, 10 min, RT, acceleration: 7, deceleration: 7.

14. Remove supernatant and resuspend the cell pellet in 5 mL of 2% FBS/PBS.

15. Centrifuge: 300× *g*, 10 min, RT, acceleration: 7, deceleration: 7.

16. Remove supernatant, resuspend the cell pellet in 1 mL of HBSS, and put 2 μL in a 1.5 mL tube. Dilute cells in the tube with 198 μL of PBS and count using a Neubauer chamber.

17. Adjust cell concentration to 50 × 10^6^ cells/mL in HBSS solution. The cell suspension is ready to be injected into mice.

**C. EBV reactivation status in plasma from PBMC donors**

Plasma is isolated from 5 mL of total fresh EDTA-treated peripheral blood using the following protocol:

1. Centrifuge: 1,200× *g*, 15 min, 23 °C, acceleration: 7, deceleration: 3.

2. Carefully collect plasma (supernatant) with a 1,000 μL pipette tip so that you do not disrupt the cell pellet.

3. Transfer plasma to 1.5 mL Eppendorf tubes.

4. Store at -80 °C.

5. When ready to perform the ELISA assay, thaw plasma at RT.

6. In our study [1], donor plasma was screened for the following six EBV antibody markers: anti-viral capsid antigen (VCA) EBV IgG, anti-VCA EBV IgM, anti-VCA EBV IgA, anti-early antigen (D) [EA(D)] EBV IgG, anti-Epstein–Barr nuclear antigen 1 (EBNA-1) EBV IgG, and anti-EBNA-1 EBV IgM, although this is an optional step.

7. Dilute plasma 1:100 in dilution buffer (provided by the VIDIA kits).

8. Perform ELISA assays according to the manufacturer’s instructions (VIDIA).

9. Measure the intensity of the color reaction using a spectrophotometer/colorimeter (Tecan Spark® or equivalent) at 450 nm within 20 min of stopping the reaction. A 620–690 nm reference filter is recommended.

10. Evaluate data according to the manufacturer’s instructions.

11. The interpretation of EBV reactivation status is based on the criteria listed in Table 1.

*Note: Each reaction’s volume is 100 μL per well, and it is advisable to use duplicates for each ELISA EBV test.*

**Table 1.** Clinical interpretation of EBV reactivation status

VCA EBV			EA(D) EBV		EBNA-1 EBV		Stages of EBV infection
IgG	IgM	IgA	IgG	IgM	IgG*	IgM*	
-	-	-	-	-	-	-	Seronegative
-	+	+	-	+ or -	-	+	Primoinfection (early phase)
+ low avidity	+	+	+ or -	+ or -	-	+	Primoinfection
	+	-	+ or -	+ or -	-	+	
	-	+	+ or -	+ or -	-	-	
+ high avidity	+	-	+ or -	-	+	-	Suspect reactivation
	-	+	+ or -	-	+	-	
	-	-	++	-	+	-	
+ high avidity	-	-	-	-	+	-	Seropositivity without symptoms of active infection

*Findings of abnormally high levels of both IgG and IgM anti-EBNA-1 antibody classes indicate EBV anomalous reactivation and a possible autoimmune condition.

Epstein–Barr virus (EBV), viral capsid antigen (VCA), early antigen [EA(D)], Epstein–Barr nuclear antigen 1 (EBNA-1), immunoglobulin G (IgG), immunoglobulin M (IgM), immunoglobulin A (IgA).

**D. Mouse engraftment with PBMC**

1. In the animal house, place the stand, mouse restrainer, and infrared thermal lamp into the BSL-2 laminar flow cabinet for animals.

2. Turn on UV for 10 min.

*Note: You should leave the room during these 10 min to avoid UV radiation.*

**Figure 2.**
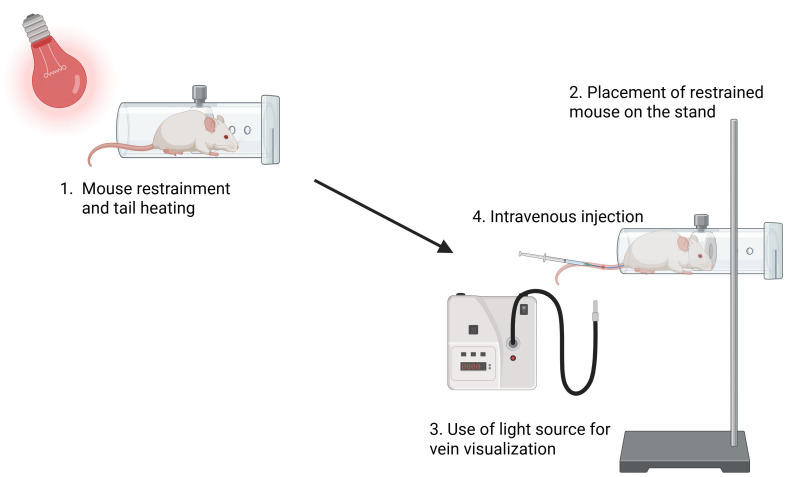
Peripheral blood mononuclear cell (PBMC) transplantation into B2m-NOG mice. A B2m-NOG mouse is placed in the restrainer, and the tail is placed close to the thermal lamp (~10 cm distance). The restrainer is secured by a stand, and the tail is sterilized with 70% ethanol using a cotton swab. Using a fiber optic light source for easier visualization of the veins, 10 × 10^6^ PBMCs are injected intravenously into a lateral tail vein.

3. Turn on the laminar flow and place the mouse cage inside the cabinet.

4. Place a mouse in the mouse restrainer and secure it with the stand.

5. Place the tail close (~10 cm distance) to the infrared thermal lamp for 30 s.

6. Using a cotton swab, gently sterilize the tail with 70% ethanol by wiping the skin toward the tail tip.

7. Use a fiber light source to locate the lateral mouse tail vein. Draw 200 μL (10 × 10^6^ cells) of resuspended cell suspension into a 1 mL insulin syringe with a 29G needle and inject slowly into the mouse tail vein (intravenous injection) (Figure 2).

**CAUTION:** This step needs a lot of stability so that the cell suspension is injected smoothly into the tail vein. Push the plunger in slowly and steadily until the required volume has been injected. If the needle is in the vein, there will be no resistance. If the needle is not in the vein, there will be resistance, and proceeding with the injection is not advised.

8. Gently remove the needle while pressing the injection site with a cotton swab to stop bleeding.

9. Gently place the mouse back into the cage. Injected mice should be weighed and monitored daily for their well-being and possible development of spontaneous clinical symptoms (see Section G).

**E. Human peripheral blood flow cytometry immunoprofiling**

1. Place 100 μL of whole blood into a 1.5 mL tube.

2. Add 1 mL of Quicklysis erythrocyte lysis buffer and incubate for 20 min at RT.

3. Centrifuge: 300× *g*, 10 min, RT, acceleration: 7, deceleration: 7.

4. Prepare antibody mastermix by mixing 2 μL of each of the following antibodies (Table 2) in a clean 1.5 mL tube.

**Table 2.** Immune cell marker antibodies for human blood

Antibody	Cell type	Working concentration
BV-510 anti-human CD45 (clone 2D1)	Pan-leukocytes	2 μg/mL
APC-Cyanine 7 anti-human CD3 (clone HIT3a)	T lymphocytes	1 μg/mL
PE-Cyanine 7 anti-human CD4 (clone A161A1)	T helper cells	2 μg/mL
PerCP anti-human CD8 (clone SK1)	T cytotoxic cells	1 μg/mL
PE anti-human CD19 (clone 4G7)	B lymphocytes	4 μg/mL
FITC anti-human CD56 (clone 5.1H11)	Natural killer cells	4 μg/mL
APC anti-human CD66b (clone G1OF5)	Neutrophils	4 μg/mL
BV421 anti-human CD14 (clone HCD14)	Monocytes	3 μg/mL

5. Remove supernatant, resuspend the cell pellet in 100 μL of PBS, and add antibody mastermix.

6. Incubate for 30 min at RT.

7. Add 1 mL of PBS and centrifuge: 300× *g*, 10 min, RT, acceleration: 7, deceleration: 7.

8. Remove the supernatant, resuspend the cell pellet in an appropriate volume of PBS (300–350 μL), and transfer to flow cytometry tubes.

9. Acquire data with a FACSMelody cell cytometer or similar [equipped with three lasers: blue, red, and violet (4B-2R-3V configuration)]. The cytometer must be able to detect the fluorochromes of the antibodies listed in Table 2.

**F. Blood sampling and flow cytometry immunoprofiling of mouse peripheral blood**

1. Before going to the animal house, place 10 μL of heparin into 1.5 mL tubes (one for each mouse).

2. In the animal house, place the stand, mouse restrainer, infrared thermal lamp, pipette, tubes, and tips into the BSL-2 laminar flow cabinet for animals.

3. Turn on UV for 10 min.

*Note: You should leave the room during these 10 min to avoid UV radiation.*

4. Turn on the laminar flow and place the mouse cage inside the cabinet.

5. Place a trained mouse (see Section D) in the mouse restrainer and secure it with the stand.

6. Place the tail close to the infrared thermal lamp (~10 cm distance).

7. Using a cotton swab, gently sterilize the tail with 70% ethanol by wiping skin toward the tail tip.

8. Use a fiber light source to locate the mouse’s tail vein. Using a fine needle, puncture the vein, starting close to the tip and progressively working toward the tail base in successive draws, and collect the blood using a pipette with a 200 μL tip.

9. Massage the tail to facilitate blood flow.

10. Repeat steps F7–8 until you collect at least 50–80 μL of blood.

11. Gently press the tail with a cotton swab to stop bleeding and place the mouse back in the cage.

12. In the laboratory, add 1 mL of Quicklysis erythrocyte lysis buffer to the tubes and incubate for 25 min at RT.

13. Centrifuge at 300× g for 10 min at RT.

14. Prepare antibody mastermix by mixing 1 μL of each of the following antibodies (Table 3) in a new 1.5 mL tube containing 100 μL of PBS for each sample (e.g., if you have 8 samples, you have to put 800 μL of PBS in the tube. Making mastermix for 2–3 extra samples is recommended).

**Table 3.** Immune cell marker antibodies for mouse blood

Antibody	Cell type	Working concentration
BV-510 anti-human CD45 (clone 2D1)	Pan-leukocytes	1 μg/mL
APC-Cyanine 7 anti-human CD3 (clone HIT3a)	T lymphocytes	0.5 μg/mL
PE-Cyanine 7 anti-human CD4 (clone A161A1)	T helper cells	1 μg/mL
PerCP anti-human CD8 (clone SK1)	T cytotoxic cells	0.5 μg/mL
PE anti-human CD19 (clone 4G7)	B lymphocytes	2 μg/mL
FITC anti-human CD56 (clone 5.1H11)	Natural killer cells	2 μg/mL
APC anti-mouse CD45 (clone 30-F11)	Pan-leukocytes (mouse)	2 μg/mL
BV421 anti-human CD14 (clone HCD14)	Monocytes	1.5 μg/mL

15. Remove the supernatant and resuspend the cell pellet in 100 μL of the antibody mastermix.

16. Incubate for 30 min at 4 °C.

17. Add 1 mL of PBS and centrifuge at 300× *g* for 10 min at 4 °C.

18. Resuspend the cell pellet in an appropriate volume of PBS (300–350 μL) and transfer to a flow cytometry tube.

19. Acquire data with a FACSMelody cell cytometer or similar cytometer [equipped with three lasers: blue, red, and violet (4B-2R-3V configuration)]. The cytometer must be able to detect the fluorochromes of the antibodies listed in Table 3.

This procedure should be performed weekly to monitor human immune cell engraftment.

**G. EAE immunization with myelin peptides**

1. Immunization is performed by subcutaneous (s.c.) injection of an emulsion containing myelin peptides, which is prepared fresh on the day of immunization using the following recipe (Table 4). The total volume of emulsion for each injection is 200 μL.

**Table 4.** Myelin peptide cocktail for induction of EAE in PBMC humanized B2m-NOG mice

Reagent	Dose/mouse	Quantity/mouse	For 10 mice
Mouse MOG35–55	100 μg (stock: 10 μg/μL)	10 μL	100 μL
Human MOG35–55	100 μg (stock: 10 μg/μL)	10 μL	100 μL
MOG1–20	100 μg (stock: 10 μg/μL)	10 μL	100 μL
MBP83–99	100 μg (stock: 10 μg/μL)	20 μL	200 μL
H37Ra *Mycobacterium tuberculosis*	400 μg	400 μg	4,000 μg
Saline	n/a *	50 μL	500 μL
FCA	100 μL	100 μL	1,000 μL
Total	200 μL	200 μL	2,000 μL**

MOG: myelin oligodendrocyte glycoprotein; MBP: myelin basic protein; FCA: Freund’s complete adjuvant.

*The saline volume is adjusted so that the final volume of the peptides + saline is 100 μL.

**CAUTION:** **The emulsion is very viscous, and there is loss during the syringe loading and injection procedures. It is recommended to make one extra dose calculated for every 4 mice.

2. Place the appropriate amount of desiccated *Mycobacterium tuberculosis* in the glass Douche homogenizer and add saline stepwise in parts of 250 µL.

**CAUTION:** The use of a protective mask is recommended.

3. Use the pestle to dissolve the *Mycobacterium tuberculosis* in the saline.

4. Transfer the mixture using a glass pipette into a 7 mL polystyrene container.

5. Add the appropriate amount of myelin peptides and mix using a vortex shaker.

6. Aliquot the mixture (maximum 700 μL) into clean 1.5 mL microcentrifuge tubes.

7. Add an equal volume of FCA to each tube (e.g., if the volume of the mixture is 500 μL, add 500 μL of FCA).

8. Sonicate in intervals (15 s on, 10 s off, RT) until the mixture becomes an emulsion. Do a short sharp spin if needed (should not exceed 800× g).

**CAUTION:** Avoid overheating the emulsion during sonication.

9. Load emulsion on 1 mL BD Plastipak syringes. Hit the loaded syringes sideways on a hard, flat surface to remove bubbles toward the opening.

10. Prepare *Bordetella pertussis* toxin at a dose of 200 ng/injection by diluting 1 μL of toxin stock solution (concentration: 0.2 μg/μL stock) in 100 μL of saline for one injection.

11. In the animal house, place all materials in the laminar flow cabinet and turn on UV for 10 min.

*Note: You should leave the room during these 10 min to avoid UV radiation.*

**Figure 3.**
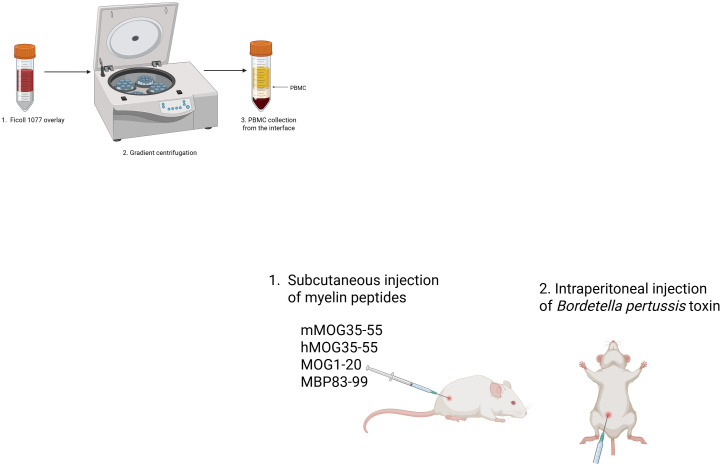
Experimental autoimmune encephalomyelitis (EAE) immunization with myelin peptides. Mice are injected subcutaneously on one hind flank close to the tail base with a Freund’s complete adjuvant (FCA) emulsion containing mMOG35–55, hMOG35–55, MOG1–20, and MBP83–99 (100 μg each peptide). Mice are also injected intraperitoneally with *Bordetella pertussis* (200 ng) on the day of immunization and 48 h later.

12. Turn on the laminar flow and place the mouse cage inside the cabinet.

13. Hold the mouse facing down, shave the hair on one hind flank close to the tail base, and inject 200 μL of emulsion subcutaneously (s.c.) at this site (Figure 3).

14. Hold the mouse abdomen up and inject 100 μL of *Bordetella pertussis* toxin intraperitoneally (i.p.). This injection should be repeated 48 h later.

15. Gently place the mouse back in the cage.

16. Mice should be weighed and monitored daily for their overall mobility and well-being and for the development of any spontaneous clinical symptoms typical of EAE (using scores described in Table 5, with 0.5 gradations representing intermediate scores), neurological dysfunction, or graft-versus-host disease (GvHD) (see Papazian et al. [1]).

**Table 5.** Clinical scoring of EAE

Score	Clinical Symptoms
0	Normal
1	Limp tail
2	Hind limb weakness
3	Hind limb paralysis
4	Forelimb paralysis
5	Moribund or dead

**Figure 4.**
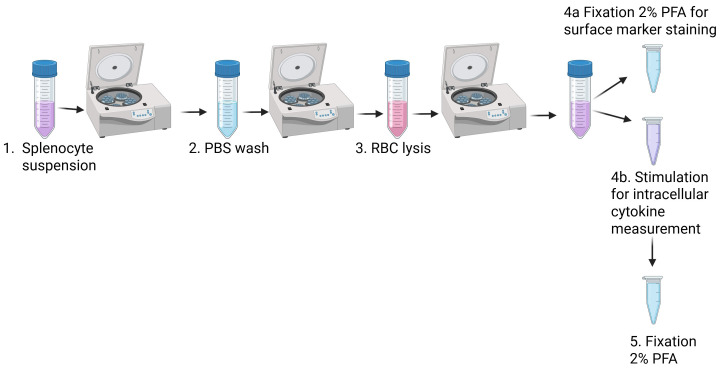
Splenocyte isolation. Spleens are placed on a Petri dish, dissociated, and transferred to a 50 mL tube and centrifuged. They are washed with PBS, centrifuged, and resuspended in Gey’s solution for red blood cell (RBC) lysis. They are centrifuged and resuspended in RPMI. One part is fixed for surface staining, and another part is stimulated for intracellular cytokine measurement and fixed afterward.

**H. Mouse splenocyte isolation (summarized in Figure 4)**

1. Humanely euthanize a mouse by cervical dislocation.

2. Remove the spleen and place it on a 94 × 16 mm Petri dish containing 5 mL of RPMI. Transfer the Petri dishes into a BSL-2 laminar flow cabinet for mouse cells. All procedures should be performed inside the BSL-2 laminar flow cabinet until the cells are fixed.

3. Dissociate the spleen using the rubber seal end of a 5 mL syringe plunger.

4. Transfer the splenocyte suspension into a 50 mL tube. Wash the Petri dish with an additional 5 mL of RPMI and add the wash to the cells.

5. Centrifuge at 300× *g* for 10 min at 4 °C.

6. Remove supernatant and resuspend the cell pellet in 10 mL of PBS.

7. Centrifuge at 300× *g* for 10 min at 4 °C.

8. Remove the supernatant and resuspend the cell pellet in 5 mL of Gey’s Solution. Leave at RT for 5 min.

9. Add 10 mL of RPMI and pass the cell suspension through a 70 μm cell strainer into a new 50 mL tube.

10. Centrifuge at 300× *g* for 10 min at 4 °C.

11. Remove the supernatant and resuspend the cell pellet in 5 mL of RPMI. For cell surface marker staining, transfer 1 mL of cell suspension (containing at least 10 × 10^6^ cells) into a clean 1.5 mL microcentrifuge tube for fixation. For intracellular cytokine measurement by flow cytometry, transfer 2 mL of cell suspension (containing at least 20 × 10^6^ cells) into a clean 15 mL tube and proceed to Section I.

12. Centrifuge at 300× *g* for 10 min at 4 °C.

13. Remove the supernatant and resuspend the cell pellet in 1 mL of PBS.

14. Centrifuge at 300× *g* for 10 min at 4 °C.

15. Remove the supernatant and resuspend the cell pellet in 400 μL of PBS. Slowly add 400 μL of 4% PFA under gentle agitation.

16. Incubate at 4 °C for 20 min.

17. Add 600 μL of PBS and centrifuge at 300× *g* for 10 min at 4 °C.

18. Remove the supernatant and resuspend the cell pellet in 400 μL of PBS. Store at 4 °C until further processing.

**I. Splenocyte preparation for intracellular cytokine measurement**

1. Perform steps H1–11.

2. For intracellular cytokine measurement by flow cytometry, transfer 2 mL of cell suspension (containing at least 20 × 10^6^ cells) into a clean 15 mL tube and centrifuge at 300× *g* for 10 min at 4 °C.

3. Remove the supernatant and resuspend the cell pellet in 1.5 mL of cytokine stimulation cocktail. Transfer into a clean 1.5 mL tube (Table 6).

**Table 6.** T-cell stimulation cocktail for intracellular cytokine detection by flow cytometry

Reagent	Stock concentration	Working concentration	Dilution	For 1 sample	For 10 samples
PMA	1 mg/mL	20 ng/mL	1/5,000	0.2 μL	2 μL
Ionomycin	1 mg/mL	1 μg/mL	1/1,000	1 μL	10 μL
Brefeldin A	5 mg/mL	5 μg/mL	1/1,000	1 μL	10 μL
RPMI				1.5 mL	15 mL

4. Incubate for 3 h at 37 °C with 5% CO_2_.

5. Centrifuge at 300× *g* for 10 min at 4 °C.

6. Continue as described in steps H14–18.

**J. Flow cytometry surface staining and acquisition**

1. For cell surface marker staining of fixed splenocytes, wash cells with 1 mL of PBS.

2. Centrifuge at 300× *g* for 10 min at 4 °C.

3. Prepare antibody mastermix by mixing 1 μL of each of the following antibodies (Table 7) in a clean 1.5 mL microcentrifuge tube containing 100 μL of PBS for each sample (e.g., for 8 samples, place 800 μL of PBS in the tube; making mastermix for 2–3 extra samples is recommended).

**Table 7.** Immune cell marker antibodies for mouse splenocytes

Antibody	Cell type	Working concentration
BV-510 anti-human CD45 (clone 2D1)	Pan-leukocytes	1 μg/mL
APC-Cyanine 7 anti-human CD3 (clone HIT3a)	T lymphocytes	0.5 μg/mL
PE-Cyanine 7 anti-human CD4 (clone A161A1)	T helper cells	1 μg/mL
PerCP anti-human CD8 (clone SK1)	T cytotoxic cells	0.5 μg/mL
PE anti-human CD19 (clone 4G7)	B lymphocytes	2 μg/mL
FITC anti-human CD56 (clone 5.1H11)	Natural killer cells	2 μg/mL
APC anti-mouse CD45 (clone 30-F11)	Pan-leukocytes (mouse)	2 μg/mL
BV421 anti-human CD14 (clone HCD14)	Monocytes	1.5 μg/mL

4. Remove the supernatant and resuspend the cell pellet in 100 μL of the antibody mastermix.

5. Incubate for 30 min at 4 °C.

6. Add 1 mL of PBS and centrifuge at 300× *g* for 10 min at 4 °C.

7. Remove the supernatant, resuspend the cell pellet in an appropriate volume of PBS (300–350 μL), and transfer to flow cytometry tubes.

8. Acquire data with a FACSMelody cytometer or similar [equipped with three lasers: blue, red, and violet (4B-2R-3V configuration)]. The cytometer must be able to detect the fluorochromes of the antibodies listed in Table 7.

**K. Flow cytometry intracellular staining and acquisition**

1. For intracellular staining of cytokines in fixed splenocytes, aliquot cells into two different clean 1.5 mL tubes and add 1 mL of permeabilization buffer to the cells. Incubate at RT for 15 min.

2. Centrifuge at 300× *g* for 10 min at 4 °C.

3. Prepare two different antibody mastermixes by mixing the following antibodies in two clean 1.5 mL microfuge tubes and making up to 100 μL with PBS/sample (Tables 8 and 9). Making mastermix for 2–3 extra samples is recommended.

*Note: The two antibody panels (Tables 8 and 9) can be collapsed into one by using another fluorochrome for either IFN-γ or IL-17A.*

**Table 8.** Antibodies for intracellular detection of IFN-γ in T cells by flow cytometry

Antibody	Quantity/sample	Working concentration
APC-CD4 (clone RPA-T4)	1 μL	0.5 μg/mL
PerCP-CD8 (clone SK1)	1 μL	0.5 μg/mL
PE-IFN-γ (clone B27)	15 μL	7.5 μg/mL

**Table 9.** Antibodies for intracellular detection of IL-17A in T cells by flow cytometry

Antibody	Quantity/sample	Working concentration
APC-CD4 (clone RPA-T4)	1 μL	0.5 μg/mL
PerCP-CD8 (clone SK1)	1 μL	0.5 μg/mL
PE-IL-17A (clone eBio64DEC17)	2 μL	1 μg/mL

4. Remove the supernatant and resuspend the cell pellet in 100 μL of the appropriate flow cytometry antibody mastermix.

5. Incubate for 30 min at 4 °C.

6. Add 1 mL of PBS and centrifuge at 300× *g* for 10 min at 4 °C.

7. Remove the supernatant, resuspend the cell pellet in an appropriate volume of PBS (300–350 μL), and transfer to flow cytometry tubes.

8. Acquire data with a FACSCalibur cytometer or similar (the cytometer must be equipped with two lasers, blue and red, and must be able to detect the fluorochromes of the antibodies listed in Tables 8 and 9).

## Data analysis

**Figure 5.**
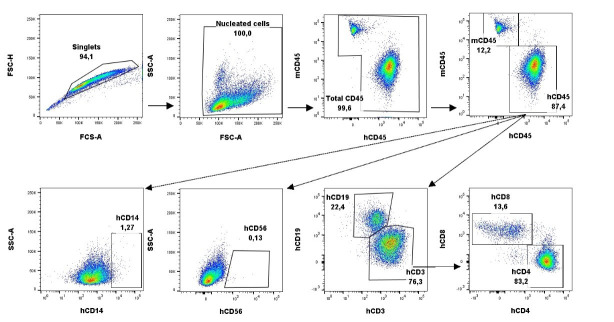
Gating strategy for the analysis of humanized mouse peripheral blood and spleen. FCS files are dragged into the FlowJo software. Singlets are gated based on size using forward scatter area (FSC-A) and forward scatter height (FSC-H). Nucleated cells are gated based on size and granularity using FSC-A and side scatter area (SSC-A). Total leukocytes are gated on mouse CD45 (mCD45) and human CD45 (hCD45). Monocytes and natural killer cells (NK) are chosen out of the hCD45 gate using human CD14 (hCD14) and human CD56 (hCD56), respectively. T and B cells are chosen out of the hCD45 gate as hCD3+ and hCD19+, respectively. T helper cells and T cytotoxic cells are further identified as hCD4+ and hCD8+, respectively, out of the hCD3+ gate. Images from the spleen from one representative mouse.

**Figure 6.**
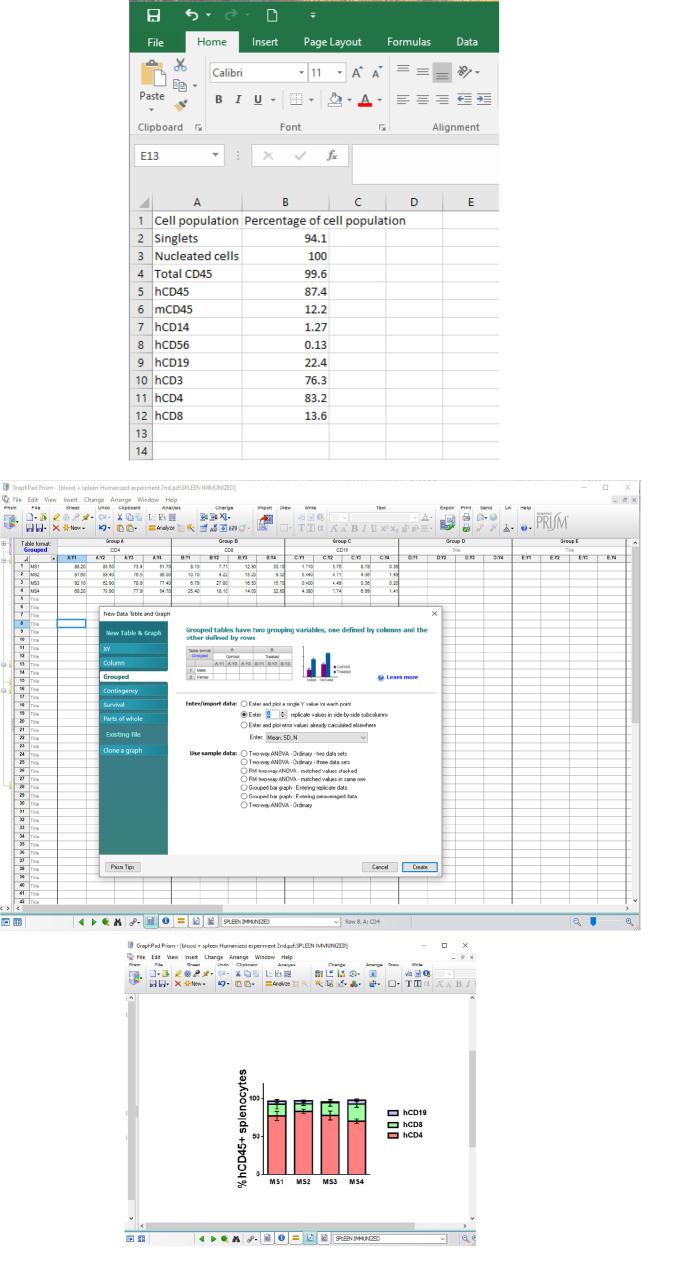
Data analysis using Microsoft Excel and GraphPad Prism. Percentages of cell populations of the FlowJo analysis are entered onto an Excel sheet (top panel) and GraphPad Prism (grouped table and the number of samples in *Enter/import data* is added) (middle panel). In this example, a stacked bars graph was chosen; the bars represent the mean with standard error of the mean (SEM) (bottom panel).

All flow cytometry data are acquired as flow cytometry standard (FCS) files and analyzed using the FlowJo software (Tree Star, Inc., V.10), using the suggested gating strategy shown in Figure 5. Percentages of cell populations are captured by this analysis and further used for statistical analysis using Microsoft Excel and GraphPad Prism software (Figure 6). The biological replicates used in the original research paper were n = 3 mice/group or 4–5 mice/group [1]. Statistical analyses were performed using Student’s t-test (as shown in Figure 1 of [1]). Results were considered statistically significant when p ≤ 0.05.

## Validation of protocol

**Human immune system engraftment**

In our original study by Papazian et al. [1], transplanted HIS mice were monitored for human immune cell engraftment by flow cytometry analysis of small blood samples recovered from the tail vein at different time points (7, 13, 42 dpt) and the spleen at sacrifice on 42 dpt; all showed progressive engraftment by human CD45+ (hCD45+) leukocytes from Day 7 (Figure 1A from [1]; Figure 1, Figure supplement 4A from [1]). Flow cytometry analysis showed preferential expansion of hCD4+ T lymphocytes compared to hCD8+ T lymphocytes in blood and spleens of all engrafted mice, and of hCD19+ B cells in mice engrafted with PBMC from several donors ( Figure 1B, C from [1]; Figure 1, Figure supplement 4B from [1]). Notably, B-cell engraftment was best in MS patients interpreted as having suspected recent or ongoing reactivation of EBV, as determined by plasma levels of anti-EBV antibodies (Table 1 from [1]; Figure 1, Figure supplement 1C from [1]). Human immune cells other than T and B cells, notably monocytes, were undetectable in dpt 42 spleen. Analysis of cytokine production by intracellular staining showed high proportions of interferon-γ (IFN-γ)-producing hCD4+ and hCD8+ splenocytes, and IL-17A-producing hCD4+ and hCD4− splenocytes in both immunized and non-immunized mice ( Figure 1D from [1]). Functionality of the human immune system in B2m-NOG mice was validated using an ex vivo proliferation assay (CFSE dilution assay) for T-cell responses in splenocytes with the four myelin peptides used for immunization (mMOG35–55, hMOG35–55, MOG1–20, and MBP83–99), and positive myelin-specific T-cell proliferation responses were measured in mice engrafted with PBMC from DR13 MS donors, although not DR15 MS donors.

**Neurological symptoms and signs of GVHD**

In our study [1], none of the typical clinical signs of EAE or other neurological deficits were observed in any of the HIS mice in the timeframe of the study (42 days dpt). It is probable that the failure of human monocyte engraftment in the B2m-NOG model is the main reason for this, since monocyte infiltration of the CNS is necessary for blood–brain barrier (BBB) disruption and the onset of clinical symptoms in EAE. Instead, we monitored disease severity by neuropathological analysis and quantitative measurement of T-cell infiltration of CNS tissues (see below). Mild symptoms of GVHD, specifically fur ruffling and reduced mobility, were recorded in a few HIS mice, independent of group, close to the time of sacrifice.

**Neuropathological analysis**

To determine whether human immune cells enter CNS tissues in PBMC humanized B2m-NOG mice, in our study [1], we performed immunohistochemical analyses of serial sections from brain ( Figure 2 from [1]) and spinal cord ( Figure 3 from [1]) recovered from non-immunized HIS mice, and brain (Figure 4 from [1]) and spinal cord (Figure 5 from [1]) from EAE-immunized HIS mice at sacrifice using marker antibodies for human and mouse immune cells as well as for mouse microglia and astrocytes. To monitor GVHD development, we performed standard histological analyses of lung and liver, two main tissue targets of GVHD (Figure 2, Figure supplement 1 from [1]). Analysis showed that EAE immunization increased infiltration of brain and spinal cord by human CD4+ and CD8 T cells compared to non-immunized mice, and that PBMC mice from DR15 MS donors showed more severe brain and spinal cord lesions than PBMC mice from DR15 healthy and DR13 MS donors (Figures 2–5 from [1])

## General notes and troubleshooting

**General notes**

We advise that human blood donors be pre-screened for pathogenic viruses so that fresh blood can be safely handled in a BSL-2 biosafety cabinet.

**Troubleshooting**

**Technical problems**

1. Insufficient numbers of PBMCs isolated from 50 mL of fresh peripheral blood for engraftment of large groups of mice.

Possible cause: The PBMC yield varies between individual blood donors.

Solution: Increase the size of the blood sample or use less than 10 × 10^6^ cells to transplant per mouse. Immune cell reconstitution can be achieved even with less (1 × 10^6^) PBMCs, as described before [23].

2. Splenocytes isolated from HIS mice are not sufficient for flow cytometry analysis.

Possible cause: Spleens in engrafted B2m-NOG mice can be small in size, containing 30–40 × 10^6^ splenocytes.

Solution: Use more cells (at least 10–15 × 10^6^) for cell stimulation and fixation.

3. Tail vein collapse and inability to perform i.v. injection.

Solution: An alternative route of administration is advised, such as intraperitoneal (i.p.), as previously described [23], or retro-orbital, although we have not tested these alternative methods.

**Limitations of the model**

1. Limited human monocyte engraftment and demyelination: B2m-NOG mice were used in this study as a state-of-the-art severely immunodeficient mouse strain capable of ready engraftment by human PBMC without prior irradiation. Particularly, the lack of MHC class I molecules (B2m) facilitates increased CD4:CD8 ratios, important for the study of autoimmune diseases like MS, and provides a longer time window before the onset of xenogeneic GVHD [19]. A main problem encountered in our study [1] was limited human monocyte/macrophage engraftment, which likely underlies the lack of CNS demyelination even in the presence of severe human T-cell infiltration of the mouse CNS tissues. Therefore, the main limitations of this model that require further development are poor monocyte engraftment and lack of demyelination, lymph node organization, and IgG responses [1].

2. Variability of engraftment immune profiles between different PBMC donors. Immune and neuropathological analyses revealed significant differences in the human immune cell profiles and CNS pathology between groups of mice engrafted with PBMC from different donors. This is to be expected, considering the heterogeneity in variables such as age, sex, genotype, and health between different human donors. We consider this variation to be an advantage, rather than a disadvantage of the particular model, that will allow for personalized approaches to studies of human immune system function, susceptibility to particular immune conditions, and responses to drugs.

3. The sample size of the PBMC donors used in our study did not allow us to make conclusions about whether variables such as age, sex, or HLA genotype affect engraftment success. This will need to be established by further studies using initially PBMCs from healthy donors recruited according to strict inclusion criteria.
